# Evaluation of the Accuracy of Probabilistic Record Linkage Across Sociodemographic Categories in 4 Databases: Exploratory Study

**DOI:** 10.2196/78622

**Published:** 2026-02-26

**Authors:** Cristina Barboi, Fangqian Ouyang, Lauren Lembcke, Andrew Martin, Ashley Griffith, Katie S Allen, Xiaochun Li, Huiping Xu, Shaun J Grannis

**Affiliations:** 1 Center for Biomedical Informatics, Regenstrief Institute, 101 W 10th Street, Indianapolis, IN, 46202, United States, 1 262 8538872, 1 317-274-0275; 2 Department of Anesthesiology, Indiana University School of Medicine, Indianapolis, IN, United States,; 3 Department of Biostatistics and Health Data Science, School of Medicine, Indiana University, Indianapolis, IN, United States; 4 Regenstrief Data Services, Regenstrief Institute, Indianapolis, IN, United States; 5 Department of Health Policy and Management, Indiana University, Indianapolis, IN, United States; 6 Department of Family Medicine, Indiana University School of Medicine, Indianapolis, IN, United States

**Keywords:** record linkage, data quality, sociodemographic characteristics, matching algorithms, matching accuracy

## Abstract

**Background:**

Accurate patient record linkage is essential for clinical care, health information exchange, research, and public health surveillance. However, linkage accuracy may vary across demographic groups due to differences in data completeness, quality, and the structural factors underlying how demographic information is captured.

**Objective:**

This study aimed to explore whether probabilistic patient matching accuracy varies by age, sex, race, and ethnicity and to identify potential sources of bias that may influence matching performance.

**Methods:**

We used 4 Indiana data sources—the Indiana Network for Patient Care, Newborn Screening, Social Security Administration Death Master File, and Marion County Public Health Department—and applied a modified Fellegi-Sunter probabilistic linkage algorithm accommodating missing data under a missing at random assumption. Gold standard match status was established through dual manual review with adjudication. For each dataset, matching sensitivity, positive predictive value, and *F*_1_-scores were estimated and stratified by age, sex, race, and ethnicity. Data completeness, distinct value ratio, and Shannon entropy were assessed to characterize data quality. Ninety-five percent bootstrap CIs were used to assess significance.

**Results:**

The algorithm-matching *F*_1_-score was greater than 0.82 for all age strata, ranging from 0.88 to 0.97 for sex, 0.85 to 0.99 for race, and 0.88 to 0.99 for ethnicity. Sensitivity ranged from 0.70 to 0.97 across age strata, 0.76 to 0.97 across sex, 0.85 to 0.99 across race, and 0.85 to 0.989 across ethnicity. Lower sensitivity and *F*_1_-scores were consistently observed in strata with greater missingness or discordance, particularly in Newborn Screening and Social Security Administration Death Master File. Race and ethnicity exhibited the highest missingness and lowest informational diversity, coinciding with the largest declines in accuracy. Shannon entropy and distinct value ratio varied across demographic groups and were strongly associated with performance, indicating that both low and excessively high informational diversity can impair matching.

**Conclusions:**

Probabilistic patient matching accuracy is not uniform across demographics and is strongly influenced by data quality and completeness. Although overall matching performance, as assessed by the *F*_1_-score, remained above 0.8, it varied across datasets when stratified by sociodemographic characteristics. Sociodemographic data missingness is associated with lower matching accuracy, raising equity and ethical concerns for clinical, research, and public health applications. Routine demographic-stratified evaluations of matching accuracy, improved standardization of sociodemographic data, and fairness-aware linkage methods are essential to prevent the amplification of structural inequities in linked health datasets.

## Introduction

### Clinical Significance

Patients receive care in many settings along their health care journey; however, their information is often stored in separate, organization-specific electronic health records. Each encounter—whether in a hospital, outpatient setting, laboratory, or public health department—generates new patient data that may not be accurately linked across health systems. Errors in patient identification can lead to duplicate or incomplete health records, jeopardizing patient safety and wasting health care time and resources.

Duplicate records occur in approximately 5% to 10% of hospital electronic health records, with up to 92% resulting from human error during registration [1]. These errors increase health care costs—averaging US $1950 per inpatient stay and more than US $800 per emergency department visit [2]. Overwriting one patient’s record with another’s data introduces additional safety risk and can compromise clinical care. Identification inaccuracies also contribute to financial inefficiencies—approximately 33% of all denied insurance claims are linked to patient identification errors, costing the US health care system over US $6 billion annually [3]. Collectively, record linkage errors affect patients, providers, and payers and have both clinical and economic consequences.

### Background

Accurate linkage of individual patient records is essential for integrating information across encounters and creating reliable, longitudinal data resources. Record linkage supports basic intrasystem tasks—such as matching laboratory results to the correct patient—as well as more complex information exchange across health care organizations. Several methodologies exist for matching health care records, including (1) deterministic rule–based approaches, (2) probabilistic methods that assign match weights or scores, (3) machine learning–based models, and (4) hybrid approaches combining multiple techniques [4,5]. Thus far, no single approach has emerged as universally superior [6].

Matching algorithms rely on identifiers such as name, date of birth, social security number, address, and their performance depends on how complete, accurate, and consistently formatted these fields are [6]. Consequently, effective patient linkage depends on both data quality [7,8] and the robustness of the matching method [9]. In this context, data quality reflects completeness—a populated field rather than missing—and correctness, the concordance of field values across datasets. Data quality is well established as foundational to reliable analytics and decision-making [10-12]. Structural and organizational factors can introduce systematic biases, and linkage errors arise when information is missing, incorrect, or inconsistent, leading to (1) missed matches (records belonging to the same individual are not linked) or (2) false matches (records from different individuals are erroneously linked) [13]. Linkage errors do not occur at equal rates across populations. Patients from ethnic minorities, particularly those with naming conventions not aligned with Western standards, experience higher rates of missed or incorrect matches [14].

Additionally, missing demographic data are more prevalent among specific populations due to social, linguistic, or contextual factors [15]. Sociodemographic characteristics, such as age, ethnicity, race, social vulnerabilities, geographical setting, and health status, have been associated with lower data completeness and higher error rates [15,16]. However, the degree to which matching performance itself varies across these factors has not been systematically examined. Given the documented disparities, identifying and understanding sociodemographic patterns associated with linkage errors is essential for recognizing and mitigating bias in patient record linkage.

### Study Objective

Unlike deterministic record-matching approaches that require exact agreement across fields, probabilistic methods assign probabilities based on weighted combinations of multiple attributes. This design makes them more sensitive to variations in data quality and demographic characteristics and therefore relevant for studying potential algorithmic bias. The Fellegi-Sunter (FS) framework is one of the most widely used and well-characterized probabilistic methods in operational US health care environments. In this study, we selected the FS framework to evaluate a core linkage approach representative of real-world practice. Although many operational systems incorporate additional deterministic or hybrid rules, such configurations are highly customized and not easily generalizable. Focusing on the probabilistic foundation most commonly deployed allows us to assess how demographic factors may influence match accuracy. Accordingly, the objective of this study was to explore whether probabilistic patient matching accuracy varies by age, sex, race, or ethnicity and to identify potential data-related sources of bias that may influence matching performance.

## Methods

### Patient Demographic Data

This study used 4 Indiana-based patient demographic data sources. First, the Indiana Network for Patient Care (INPC), a statewide health information exchange containing over 47 million registration records across more than 100 clinical data sources, including emergency department visits, hospital admissions, and large outpatient health care clinics from across the state [17]. Second, Newborn screening (NBS) data, consisting of demographic data derived from health information exchange-wide Health Level 7 messaging for children aged under 12 months. This dataset includes limited newborn screening information collected within 5 days of birth, mandated by Indiana law. Third, Social Security Administration Death Master File (SSA), containing records of individuals issued a Social Security number whose deaths were reported to the SSA. Fourth, Marion County Public Health Department (MCPHD), the county’s infectious disease reporting database. Marion County is Indiana’s largest county, with a population of just over 966,000.

### Generation of Patient Record Pairs

This study leveraged analytic datasets from previous studies on patient matching and record linkage [2,18]. The FS model was chosen for this project due to its widespread use and flexible maximum-likelihood framework. To accommodate missing data in real-world settings, we applied an established modification of the FS model under the missing at random (MAR) assumption [19,20]. Under the MAR assumption, missing fields are handled using the full-information likelihood approach, which incorporates all available data while excluding only the specific missing fields, assuming conditional independence across variables. Using the modified FS probabilistic algorithm [21], we identified matches and nonmatches across four dataset pairs: (1) INPC to INPC (labeled as INPC), (2) NBS to NBS (labeled as NBS), (3) INPC to SSA (labeled as SSA), and (4) INPC to MCPHD (labeled as MCPHD). Matching variables included medical record number, first and last name, middle initial, sex, telephone number, address, ZIP code, social security number, and components of the date of birth.

### Manual Review of Record Pairs

To construct a gold standard reference, we randomly sampled record pairs from each dataset for manual review. For each sampled pair, complete reference data from both records were provided to the reviewers to support accurate adjudication [2,22]. Using a balanced, incomplete block design, 2 independent reviewers assessed each pair’s match status [20], while a third reviewer resolved any discrepancies. A total of 62,000 pairs were manually reviewed: 15,000 INPC, 15,000 NBS, 16,500 SSA, and 15,500 MCPHD. As the reviewers’ identities were inconsistent during the assessment of pairs’ match status, Cohen κ no longer applied; therefore, the overall agreement rate was calculated. The detailed methodology and outcomes of this manual review process were described previously [22]. For the current analysis, all gold standard referential pairs were stratified by race, ethnicity, sex, and age.

### Analysis

We conducted an exploratory analysis of sociodemographic variables—age, sex, race, and ethnicity—evaluating each variable independently for each record pair. Record pairs were assigned to a given stratum (eg, *Asian*) only when both records contained nonmissing, concordant values for that variable. *Missing* data (one or both fields absent) were distinguished from *discordant* data (both fields present but disagreeing). Pairs with discordant values for a specific variable were excluded from the performance calculations for that variable’s stratum. However, they could contribute to analyses of other sociodemographic strata where their values were concordant. As a single record could appear in multiple pairings, individuals may be represented more than once. Missing data were addressed using the MAR-modified FS model, which uses all available information without defaulting to disagreement for missing values. Previous research shows that this modification improves linkage accuracy and *F*_1_-scores compared with traditional missing data handling [18]. The linkage algorithm was applied separately within each sociodemographic stratum, supporting the MAR assumption within these more homogeneous groups. While MAR is reasonable in many operational contexts, data can be missing not at random (MNAR). Evaluating MNAR scenarios would require alternative missingness models and sensitivity analyses, which we identified as an area of future work.

### Accuracy of Probabilistic Record Linkage

For each stratum, we compared the algorithm’s declared match status with the gold standard referential reviewer classification. We estimated sensitivity, positive predictive value (PPV), and *F*_1_-score, along with 95% CI derived from 1000 bootstrap samples, using the percentile method. CIs were reported to indicate the precision of estimated performance measures rather than to support formal hypothesis testing or draw inferential conclusions. Given the exploratory nature of this analysis, no corrections for multiple comparisons were applied.

### Data Quality Assessment

To evaluate data quality across sociodemographic strata, we calculated (1) missing data ratio (MDR), measures *data completeness* as percent of records in which a field was missing; (2) distinct value ratio (DVR), measures *data uniqueness* as number of unique values for a field divided by the number of nonmissing records; and (3) Shannon entropy (SE), a measure of *information content*, increasing when values are numerous and evenly distributed [8,23].

### Ethical Considerations

This research was approved by the Indiana University Institutional Review Board (IRB 170375536) under category 5 (research involving materials collected for nonresearch purposes), which granted a waiver for informed consent. The study used retrospective data collected during routine care, with no contact with individuals and minimal risk to privacy or welfare. Although datasets contained identifiable information, data were stored on Health Insurance Portability and Accountability Act–compliant remote servers and accessed only by authorized personnel via encrypted connections. All project-related data were managed according to the university security protocols and institutional storage infrastructure. As confidentiality protections were deemed adequate, no ongoing institutional review board monitoring beyond the approval determination was required. All accessed data were governed through data sharing agreements with each contributing entity.

## Results

### Reviewers’ Agreement on Match Status

During the creation of the referential gold standard–linked datasets, reviewers agreed on the match status for 96.1% of record pairs; the remaining 3.9% required adjudication by a third reviewer due to disagreement.

### Data Quality Assessment

Table 1 summarizes the sociodemographic characteristics of the record pairs included in the referential gold standard datasets. For each variable, the table reports the number of record pairs (N) with nonmissing values in the INPC, NBS, SSA, and MCHD datasets; variables not collected in a dataset are marked “n/a.” Notably, race and ethnicity are absent in the SSA and MCPHD datasets; therefore, they were excluded from the analysis. In the NBS dataset, age was not assessed because all newborns were within 12 months of age.

**Table 1.** Sociodemographic characteristics of the record pairs included in the referential gold standard datasets.

Sociodemographic variables	INPC^a^ record pairs (n=15000)	NBS^b^ record pairs (n=15,000)	SSA^c^ record pairs (n=16,500)	MCHD^d^ record pairs (n=15,500)
Age (years)				
<18	1746	N/A^e^	22	4002
18‐65	10,178	N/A	2894	10,683
>65	3035	N/A	9610	735
Sex				
Male	4867	6280	N/A	4759
Female	7368	5503	N/A	6256
Race				
White	8551	8712	N/A	N/A
Black	1323	2397	N/A	N/A
Asian	413	492	N/A	N/A
Pacific Islander	332	334	N/A	N/A
Native American	30	47	N/A	N/A
Ethnicity				
Hispanic	275	1214	N/A	N/A
Not Hispanic	7483	9309	N/A	N/A

a INPC: Indiana Network for Patient Care.

b NBS: newborn screening.

c SSA: Social Security Administration.

d MCHD: Marion County Health Department.

e N/A: not available.

Table 2 presents the degree of missingness and disagreement across demographic variables in the referential gold standard datasets. In the INPC dataset, ethnicity shows the highest level of missingness, followed by race. In the SSA dataset, missing values in the ethnicity, race, and sex fields are noteworthy. Within the NBS dataset, more record pairs have missing ethnicity and race values than discordant values, whereas the sex field shows more discordance than missingness.

**Table 2.** Missingness and disagreement in demographic characteristics in the referential standard–linked datasets.^a^

Sociodemographic variable and record missingness or disagreement	INPC^b^ dataset (n=15,000 pairs; 30,000 records)	NBS^c^ dataset (n=15,000 pairs; 30,000 records)	SSA^d^ dataset (n=16,500 pairs; 33,000 records)	MCPHD^e^ dataset (n=15,500 pairs; 31,000 records)
Ethnicity				
Missing	14,266	8102	33,000	31,000
Discordant	422	1398	N/A^f^	N/A
Sex				
Missing	196	320	33,000	540
Discordant	5334	6112	N/A	8430
Age				
Missing	16	N/A	52	2
Discordant	66	N/A	7896	248
Race				
Missing	8504	5062	33,000	31,000
Discordant	2060	2674	N/A	N/A

a The data reflect the raw number of individual records within a pair with missing or discordant demographic values.

b INPC: Indiana Network for Patient Care.

c NBS: newborn screening.

d SSA: Social Security Administration.

e MCHD: Marion County Health Department.

f N/A: not available.

The relative proportion of missing sex, race, and ethnicity values varies considerably across datasets, as presented in Table 3. Across datasets, the MDR ranges from 0.20 to 0.65 for race, 0.40 to 0.84 for ethnicity, and 0.003 to 0.5 for sex. As sex, race, and ethnicity each contain only a few valid categories, the DVR provides limited additional insight into data quality. SE, which generally measures the information diversity in the data, can be falsely elevated by artifacts, errors, or inconsistent encoding. SE values for sex were similar across datasets, ranging from 1.01 to 1.4, whereas the broader range observed for race and ethnicity probably reflected data quality issues rather than true variability (Table 3).

**Table 3.** Measures of data quality: missing data ratio (MDR), distinct values ratio (DVR), and Shannon entropy (SE) measured in bits.

Dataset and sociodemographic variable	MDR	DVR	SE (bits)
INPC^a^			
Sex	0.0037	0.0001	1.0117
Race	0.6171	0.0002	1.2863
Ethnicity	0.8416	0.0001	0.6863
SSA^b^			
Sex	0.5012	0.00009	1.4946
MCHD^c^			
Sex	0.0089	0.0001	1.0623
NBS^d^			
Sex	0.0058	0.0001	1.0455
Race	0.2067	0.0002	1.6530
Ethnicity	0.4002	0.0001	1.3313

a INPC: Indiana Network for Patient Care.

b SSA: Social Security Administration.

c MCHD: Marion County Health Department.

d NBS: newborn screening.

### Algorithm Matching Accuracy

**Figure 1.**
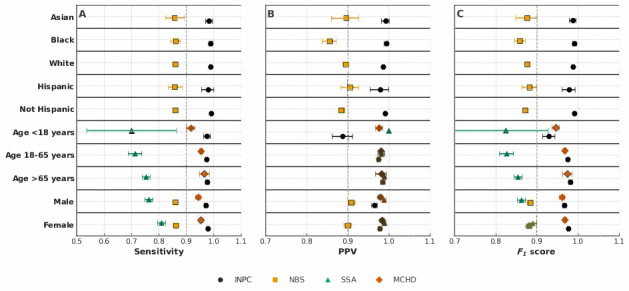
(A) Sensitivity, (B) positive predictive value (PPV), and (C) *F*_1_-score for probabilistic patient matching performance across demographic groups. Points indicate the estimated metric, and horizontal bars represent 95% CIs. Results are shown for 4 data sources. INPC: Indiana Network for Patient Care; MCHD: Marion County Public Health Department; NBS: newborn screening; SSA; Social Security Administration.

Figure 1 and Table S1 in Multimedia Appendix 1 summarize the matching accuracy and corresponding CIs across all sociodemographic strata. Across age groups, matching sensitivity ranged from 0.7 to 0.97, the PPV exceeded 0.88, and the *F*_1_-scores were greater than 0.82. When stratified by sex, sensitivity ranged from 0.76 to 0.98, PPV ranged from 0.80 to 0.98, and *F*_1_-score ranged from 0.84 to 0.97. Across race groups, the algorithm’s matching sensitivity ranged from 0.58 to 0.99, PPV ranged from 0.85 to 0.98, and *F*_1_-score ranged from 0.85 to 0.99. For ethnicity, the matching sensitivity ranged from 0.85 to 0.989, the PPV ranged between 0.88 and 0.99, and the *F*_1_-score ranged from 0.88 to 0.99. Compared with INPC and MCPHD, the SSA dataset showed significantly lower sensitivity and *F*_1_-scores across all age strata (<18 y, 18-65 y, and >65 y), as indicated by nonoverlapping 95% CIs. Similarly, for both males and females, matching sensitivity and *F*_1_-scores in SSA and NBS were significantly lower than those observed in INPC and MCPHD. In the NBS dataset, the matching sensitivity, *F*_1_-score, and PPV for Asians, Blacks, and White racial groups were lower than those in the INPC dataset, with statistically significant differences indicated by nonoverlapping 95% CIs. The same pattern was observed across Hispanic and non-Hispanic ethnicities, with all 3 performance metrics in NBS markedly lower than in INPC.

**Figure 2.**
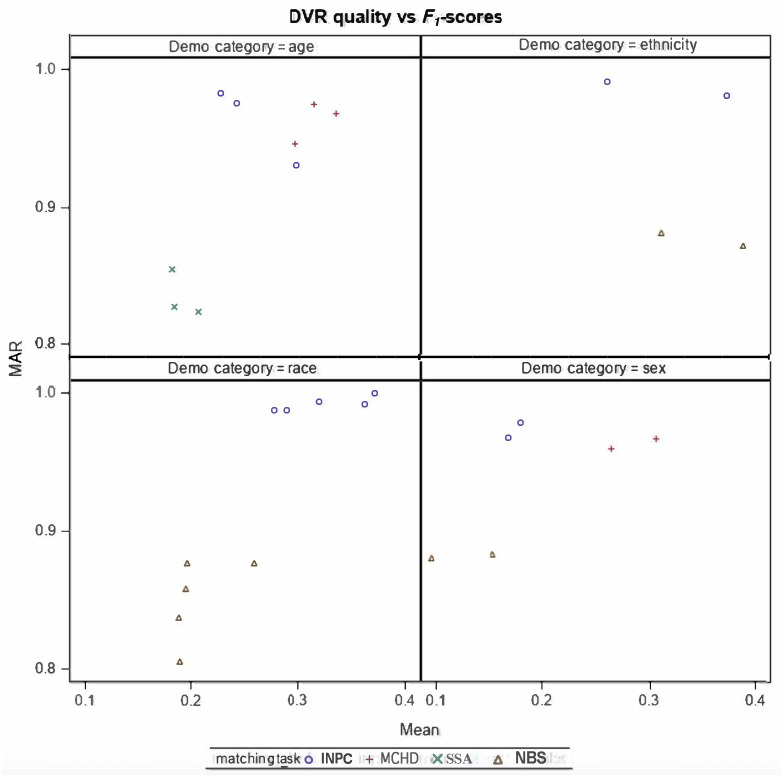
DVR quality versus *F*_1_-score plots. Mean refers to the DVR quality indicator (unitless ratio between 0 and 1), while MAR refers to the *F*_1_-score using the missing at random approach (dimensionless value between 0 and 1). DVR quality indicator$=\frac{q_{1}\left(1-ms_{1}\right)+q_{2}(1-ms_{2})+\ldots +q_{P}(1-ms_{p})}{P}$; in which missing percentage=ms_i_. DVR: distinct value ratio; INPC: Indiana Network for Patient Care; MAR: missing at random; MCHD: Marion County Public Health Department; NBS: newborn screening; SSA: Social Security Administration.

Across all datasets, the largest variability in matching performance was observed among pairs with missing or discordant field values. Data quality–performance plots (*F*_1_-score vs DVR; *F*_1_-score vs SE) show that the relationship between data quality and linkage accuracy varies across datasets, particularly for race and ethnicity in NBS and INPC (Figures2  3). For age, performance differences among datasets appear to stem more from differences in date of birth documentation formats than from true demographic variability. In INPC, the algorithm maintained consistently high performance despite variation in data quality. In contrast, the NBS dataset showed relatively similar performance across race, ethnicity, and sex groups, despite substantial differences in data quality.

**Figure 3.**
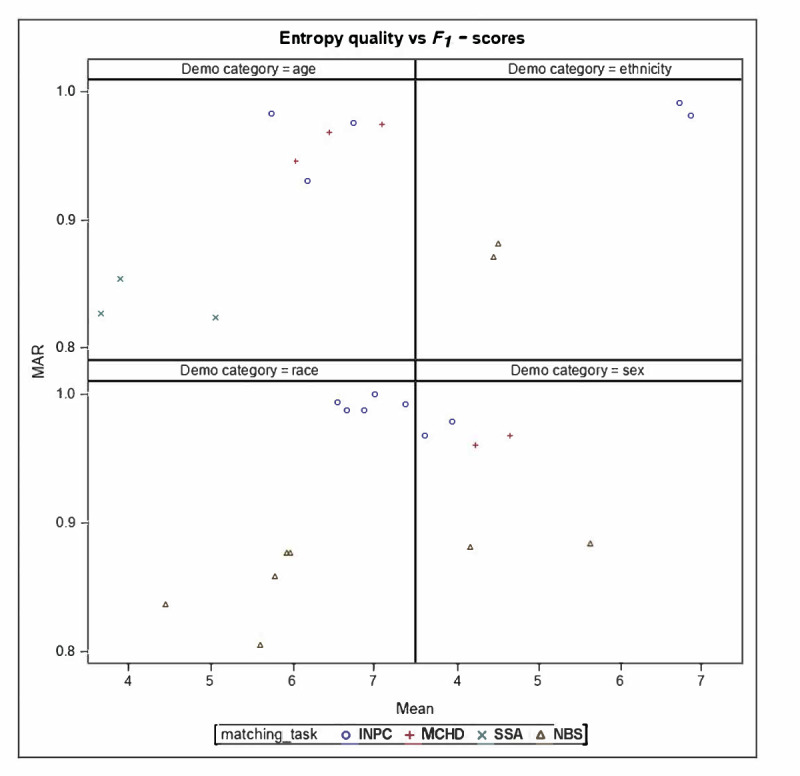
Shannon entropy quality versus *F*_1_-score plots. Mean refers to the Shannon entropy quality indicator (measured in bits), while MAR refers to the *F*_1_-score using the missing at random approach (dimensionless value between 0 and 1). Entropy quality indicator*=*$\frac{e_{1}\left(1-ms_{1}\right)+e_{2}(1-ms_{2})+\ldots +m_{P}(1-ms_{p})}{P}$*;* in which Shannon entropy=e_i_; missing percentage=ms_i_. INPC: Indiana Network for Patient Care; MAR: missing at random; MCHD: Marion County Public Health Department; NBS: newborn screening; SSA: Social Security Administration.

## Discussion

### Summary of Key Findings

This study examined whether probabilistic patient matching accuracy varies by age, sex, race, and ethnicity and sought to identify data-related sources of bias that may influence matching performance. Across 4 large, operationally relevant datasets, matching sensitivity, PPV, and *F*_1_-score varied by demographic strata, with several statistically significant differences indicated by nonoverlapping CIs. These findings show that probabilistic linkage accuracy is not demographically uniform and is influenced not only by algorithmic design but also by underlying data quality and completeness—both of which can bias matching performance.

### Variation in Data Completeness (MDR) and Its Impact on Performance

Data completeness varied across datasets. Race and ethnicity exhibited the highest levels of missingness, consistent with prior work documenting gaps in demographic data capture [24,25]. Age was generally complete except in the SSA dataset, which contained substantial discordance across linked records. These completeness patterns were directly reflected in performance: the most significant declines in sensitivity and *F*_1_-score occurred in strata with missing or inconsistent fields, and the lowest overall performance was observed in the NBS and SSA datasets, where missingness was most pronounced.

### Variation in Data Quality (DVR and SE) and Its Impact on Performance

Data quality varied across demographic fields. While the DVR showed only modest variability, SE differed substantially, particularly for sex, race, and ethnicity, indicating increased variation in the information content of these fields. Fields with lower SE or DVR values, such as those with more missing data, standardized naming conventions, or limited variability, provide less discriminative information for probabilistic algorithms, increasing the risk of false matches or missed linkages. These patterns may disproportionately affect populations with less consistent data capture, such as individuals with non-English names, hyphenated surnames, or variable address formats. Fields with more distinct values or more evenly distributed unique values have higher DVR and SE. Such fields provide more discriminative information for the linkage algorithms, improving the algorithm accuracy. The theoretical maximum entropy reflects the upper limit of information a variable could contain if all its possible values were equally likely. Comparing a variable’s observed entropy to this maximum indicates how fully the variable uses its potential informational range [8]. The plots of data quality indicators—SE and DVR—versus *F*_1_-score (Figures2  3) demonstrate a correlation between the 2 variables and warrant further exploration in future studies. Previous studies have identified similar relationships between data quality and record linkage performance [26,27].

Systematic assessment of data completeness and data quality using metrics such as the MDR, DVR, and SE extends and strengthens traditional probabilistic record linkage approaches by explicitly quantifying the informational value of matching fields and linking these properties to performance disparities. MDR captures the degree of missingness that directly reduces the discriminative power of identifiers, while DVR and SE characterize the uniqueness and information content of variables that are central to assigning match weights in probabilistic models. SE, in particular, has been used extensively to assess how effectively a variable differentiates individuals within a population, while distinct value–based metrics have been shown to correlate with linkage performance by reflecting the degree of identifier uniqueness [28,29]. Previous studies have demonstrated that linkage accuracy is highly sensitive to both missingness and variability in identifying fields, and entropy-based measures have been widely used in record linkage and data integration to evaluate information content, guide field selection, and optimize matching rules [6,28]. Similarly, distinct value–based metrics have been shown to correlate with linkage performance by reflecting the degree to which identifiers distinguish true matches from coincidental agreements [6]. Compared with deterministic linkage methods, which rely on exact or rule-based agreement and have been shown to perform poorly in settings with heterogeneous data quality or incomplete identifiers [30,31], probabilistic approaches derived from the FS framework remain the most widely used in health care and administrative data linkage due to their flexibility, interpretability, and ability to accommodate partial agreement and missing data [21,32]. However, previous evaluations of probabilistic linkage have largely focused on aggregate accuracy metrics, often obscuring subgroup-specific errors and masking the role of data quality in generating disparate performance [29].

By integrating these metrics into a probabilistic framework derived from the FS model, one of the most widely used and interpretable approaches for health care and administrative data linkage due to its ability to accommodate partial agreement and missing data [18,33], this study advances beyond deterministic and hybrid methods, which often perform poorly in settings with heterogeneous data quality or high missingness [34-36].

The framework deployed in this study improves upon these approaches by integrating a modified FS model under an MAR assumption with explicit, field-level data quality diagnostics and demographic-stratified performance assessment. This combination enables detection of prealgorithmic bias, directly links observed performance disparities to measurable data characteristics, and supports targeted mitigation strategies such as adaptive weighting, selective data cleaning, or alternative blocking schemes [13,31,36]. Compared with hybrid or black-box machine learning–based linkage methods, which may offer gains in accuracy but often lack transparency and equity assessment capabilities [37,38], this framework balances operational feasibility, interpretability, and fairness, aligning with best practices in modern record linkage and algorithmic fairness research and supporting its applicability in large-scale health information exchange environments. This integration improves transparency, interpretability, and equity assessment relative to black box or rule-based approaches, while remaining compatible with large-scale operational health information exchange systems [19,31,35,39,40].

### Potential Data-Related Sources of Bias

Multiple structural and administrative factors contribute to missing or inconsistent demographic fields, creating sources of prealgorithmic bias—bias embedded in the data before any matching occurs. Examples include self-reported or manually entered race and ethnicity, inconsistent naming conventions, and institutional practices such as assigning newborn race based on the birthing parent or the SSA’s merging of race and ethnicity into a single field [41]. These practices disproportionately affect racial and ethnic minorities and produce several forms of data-related bias. Inconsistent capture of race and ethnicity introduces measurement bias, while failure to record key demographic fields results in omitted-variable bias [42]. Biased or incomplete data can cause algorithms to reproduce and amplify inequities. When models are trained on historical datasets that reflect past discrimination, they learn these patterns and unfairly penalize underrepresented groups. Missing or sparse data for marginalized populations leads to poorer performance for those groups.

Underrepresentation bias can arise when certain groups are sparsely represented in datasets used to tune or validate linkage models [19,43-45].

Reviewer bias may further influence the construction of referential standards, particularly when adjudicators encounter unfamiliar names or naming conventions [43,46]. Finally, population-specific characteristics, such as the newborn population in the NBS dataset, whose records are newly created and collected under mandated screening workflows, can introduce additional variability in data completeness and quality.

### Clinical, Ethical, and Research Implications of Disparities in Patient Matching Accuracy

Differences in linkage accuracy across demographic groups have implications for clinical, ethical, and research practice. Disparities in patient matching accuracy can have cascading effects on health care analytics, policy, and clinical practice. When linkage accuracy is lower for underrepresented populations, their health data may be fragmented or misclassified, leading to biased reporting of outcomes and inequitable policy or clinical decisions. Record linkage errors have direct consequences for patient safety and care quality. Missed matches fragment longitudinal histories, obscuring essential information such as allergies, prior diagnoses, or medications, while false matches may merge records from different individuals, leading to inappropriate treatment, misdiagnosis, or exposure of sensitive information. These errors disproportionately affect populations whose demographic data are inconsistently captured—including racial and ethnic minorities, immigrants, individuals with unstable housing, and very young or older patients—who often experience higher missingness and discordance in key identifiers [7,47]. As a result, linkage inaccuracies can impair clinical decision-making, compromise continuity of care, and exacerbate existing health care disparities.

The ethical implications of linkage errors extend beyond clinical harm to include threats to privacy, autonomy, and fairness. Erroneous merges can reveal sensitive information and increase risks of reidentification, identity theft, or unauthorized disclosure. At the same time, missed matches can lead individuals to lose control over how their information is used across systems. These concerns are amplified for groups already affected by structural inequities, who face greater risks of linkage failure due to inconsistent or incomplete demographic data [24,25]. Such patterns mirror findings in the algorithmic fairness literature [48], which emphasizes that data completeness, representativeness, and informational balance in preprocessing must be monitored to prevent the reinforcement of structural inequities [7,44,47,49,50]. Biased model outputs can also create reinforcing feedback loops, where discriminatory decisions generate new biased data that further entrench inequity [51]. Ultimately, these mechanisms produce systematic performance disparities—algorithms perform least well for underrepresented or inaccurately recorded populations, even when no discriminatory intent exists [51]. Persistent errors can erode trust in health care organizations and public institutions, discouraging individuals from sharing information or seeking care, particularly among historically marginalized communities.

Record linkage errors also compromise the validity of research and public health surveillance. False matches introduce noise and bias estimates toward the null, whereas missed matches reduce sample size, weaken statistical power, and may undercount exposures or outcomes. These errors distort estimates of disease prevalence, treatment effectiveness, and health disparities, particularly when linkage success varies systematically across demographic groups. Unequal linkage accuracy can produce unrepresentative analytic datasets, misinform policy decisions, and result in inequitable resource allocation. As linkage errors are not randomly distributed—disproportionately affecting racial/ethnic minorities, immigrants, and individuals with unstable housing—these disparities can amplify existing inequities in population-level research and public health practice [7,47]. Accurate and consistent capture of sociodemographic information is therefore essential for evaluating algorithmic equity and ensuring fair and reliable downstream analyses [24,25]. Accurate capture of race and ethnicity is therefore essential for evaluating and ensuring equitable algorithmic performance.

### Implications for Future Work

This study’s strengths include the use of 4 large operational datasets, manually adjudicated gold standard pairs, and the integration of data quality metrics such as DVR and SE to contextualize performance variation. The findings underscore the importance of assessing linkage accuracy within demographic subgroups rather than relying on aggregate performance, which can obscure disparities.

Future work should evaluate deterministic and hybrid linkage approaches to determine whether demographic disparities persist across algorithm types. Fairness-aware linkage methods and improved demographic data capture—especially standardized race and ethnicity fields—represent promising mitigation strategies. Simulation-based analyses of MNAR mechanisms may provide insight into bias introduced by unobserved missingness. Further research should quantify the relationship between measures of data quality and the performance of the matching algorithm. Finally, research should explore the downstream consequences of linkage disparities for surveillance metrics, risk prediction models, and health equity analyses.

### Limitations

This study has several limitations. First, missing demographic data for race and ethnicity prevented comparisons of linkage performance across all race categories. Although current reporting guidance includes categories such as Native American and Pacific Islander, the small number of records in these groups precluded subgroup analyses. Sex-stratified analyses were similarly constrained by limited availability in some datasets.

Second, all data were drawn from a single US state, which may limit generalizability. Nonetheless, the inclusion of heterogeneous sources—particularly a statewide health information exchange aggregating data from more than 100 clinical organizations—supports broader applicability across diverse health system environments. Although these datasets originate in Indiana, the observed disparities in probabilistic linkage accuracy likely reflect fundamental issues of data completeness, standardization, and attribute weighting that are common across health systems. Replicating this work in multistate or international settings will be important to confirm broader relevance and to guide equity-focused improvements in patient matching methods.

Third, missing data were addressed using a modified FS model under a MAR assumption. We did not evaluate MNAR mechanisms, which may introduce additional forms of linkage bias. Future work will examine MNAR processes to characterize better and mitigate these effects.

Finally, jurisdiction-specific workflows (eg, newborn screening processes in the NBS dataset) may influence data availability and structure, potentially limiting comparability with datasets from other health systems.

### Conclusions

This study found statistically significant differences in probabilistic patient matching accuracy across age, sex, race, and ethnicity. These disparities were closely aligned with variation in data completeness, uniqueness, and informational richness across datasets, demonstrating that matching performance is shaped as much by data quality as by algorithm design. Datasets with greater missingness, discordance, or low information diversity, particularly in race and ethnicity fields, consistently showed lower sensitivity and *F*_1_-scores. This underscores the critical role of data quality in linkage performance. Such deficits in data quality and completeness introduce prealgorithmic bias, including underrepresentation bias, which can directly affect the accuracy of probabilistic matching.

As linkage errors disproportionately affect groups with poorer or less standardized demographic data, these disparities raise important clinical, public health, and ethical concerns. Fragmented records, misclassified cases, and biased population estimates can perpetuate inequities in care delivery, surveillance, and policy. Routine demographic-stratified evaluations of matching accuracy are therefore essential to detect and mitigate algorithmic bias. Future work will focus on improving the capture and standardization of sociodemographic data, developing fairness-aware linkage methods, and assessing bias from records with missing or discordant fields.

When implementing patient matching systems, performance should be monitored across subpopulations to identify prealgorithmic bias, characterize which groups are at risk, and assess the potential severity of downstream harms. Policymakers should advocate for consistent frameworks to assess matching algorithms, establish accountability mechanisms, and standardize the collection of race, ethnicity, and other important demographic factors. Improving the quality of demographic data and monitoring equity in linkage performance are critical to ensure that linked health data support fair, reliable, and inclusive clinical, research, and public health decision-making.

## Supplementary material

10.2196/78622 Multimedia Appendix 1 Matching performance parameters, stratified by sociodemographic groups across all datasets.

## Abbreviations

DVR: distinct value ratio
FS: Fellegi-Sunter
INPC: Indiana Network for Patient Care
MAR: missing at random
MCPHD: Marion County Public Health Department
MDR: missing data ratio
MNAR: missing not at random
PPV: positive predictive value
SE: Shannon entropy
