# Supplementary material for: Evaluation of the Accuracy of Probabilistic Record Linkage Across Sociodemographic Categories in 4 Databases: Exploratory Study
Source: JMIR Form Res. 2026 Feb 26;10:e78622. doi: 10.2196/78622 (PMC12945093; doi:10.2196/78622)
Supplement: Multimedia Appendix 1 [file formative-v10-e78622-s001.docx]

| **Sociodemographic variable** | **Matching**  **performance parameter** | **Dataset** | **Value Confidence**  **interval** | | **Sociodemographic Matching variable performance**  **parameter** | | **Dataset** | **Value** | **Confidence Interval** |
| --- | --- | --- | --- | --- | --- | --- | --- | --- | --- |
| **Asian** | **Sensitivity** | **INPC** | 0.983 | 0.970- 0.995 | **<18 years** | **Sensitivity** | **INPC** | 0.977 | 0.964- 0.989 |
|  | **Sensitivity** | **NBS** | 0.858 | 0.824- 0.892 |  | **Sensitivity** | **NBS** | n/a | n/a |
|  | **Sensitivity** | **SSA** | n/a | n/a |  | **Sensitivity** | **SSA** | 0.700 | 0.536-0.864 |
|  | **Sensitivity** | **MCHD** | n/a | n/a |  | **Sensitivity** | **MCHD** | 0.917 | 0.902- 0.931 |
| **Asian** | **PPV** | **INPC** | 0.992 | 0.981- 1.000 | **<18 years** | **PPV** | **INPC** | 0.887 | 0.861-0.911 |
|  | **PPV** | **NBS** | 0.895 | 0.860-0.926 |  | **PPV** | **NBS** | n/a | n/a |
|  | **PPV** | **SSA** | n/a | n/a |  | **PPV** | **SSA** | 1.000 | 1.000 |
|  | **PPV** | **MCHD** | n/a | n/a |  | **PPV** | **MCHD** | 0.976 | 0.967-0.984 |
| **Asian** | **F-score** | **INPC** | 0.988 | 0.979-0.995 | **<18 years** | **F-score** | **INPC** | 0.930 | 0.914-0.944 |
|  | **F-score** | **NBS** | 0.876 | 0.848-0.900 |  | **F-score** | **NBS** | n/a | n/a |
|  | **F-score** | **SSA** | n/a | n/a |  | **F-score** | **SSA** | 0.824 | 0.698- 0.927 |
|  | **F-score** | **MCHD** | n/a | n/a |  | **F-score** | **MCHD** | 0.946 | 0.937-0.954 |
| **Black** | **Sensitivity** | **INPC** | 0.990 | 0.984- 0.996 | **18-65 years** | **Sensitivity** | **INPC** | 0.975 | 0.970- 0.979 |
|  | **Sensitivity** | **NBS** | 0.861 | 0.843-0.878 |  | **Sensitivity** | **NBS** | n/a | n/a |
|  | **Sensitivity** | **SSA** | n/a | n/a |  | **Sensitivity** | **SSA** | 0.714 | 0.689- 0.738 |
|  | **Sensitivity** | **MCHD** | n/a | n/a |  | **Sensitivity** | **MCHD** | 0.955 | 0.949-0.961 |
| **Black** | **PPV** | **INPC** | 0.994 | 0.988- 0.998 | **18-65 years** | **PPV** | **INPC** | 0.975 | 0.971-0.979 |
|  | **PPV** | **NBS** | 0.856 | 0.838-0.872 |  | **PPV** | **NBS** | n/a | n/a |
|  | **PPV** | **SSA** | n/a | n/a |  | **PPV** | **SSA** | 0.982 | 0.973 – 0.990 |
|  | **PPV** | **MCHD** | n/a | n/a |  | **PPV** | **MCHD** | 0.981 | 0.976-0.985 |
| **Black** | **F-score** | **INPC** | 0.992 | 0.988-0.996 | **18-65 years** | **F-score** | **INPC** | 0.975 | 0.972-0.978 |
|  | **F-score** | **NBS** | 0.859 | 0.845-0.871 |  | **F-score** | **NBS** | n/a | n/a |
|  | **F-score** | **SSA** | n/a | n/a |  | **F-score** | **SSA** | 0.827 | 0.809-0.843 |
|  | **F-score** | **MCHD** | n/a | n/a |  | **F-score** | **MCHD** | 0.968 | 0.964-0.971 |
| **White** | **Sensitivity** | **INPC** | 0.988 | 0.985 – 0.991 | **>65 years** | **Sensitivity** | **INPC** | 0.978 | 0.971- 0.984 |
|  | **Sensitivity** | **NBS** | 0.859 | 0.850 -0.868 |  | **Sensitivity** | **NBS** | n/a | n/a |
|  | **Sensitivity** | **SSA** | n/a | n/a |  | **Sensitivity** | **SSA** | 0.753 | 0.738-0.767 |
|  | **Sensitivity** | **MCHD** | n/a | n/a |  | **Sensitivity** | **MCHD** | 0.966 | 0.948-0.984 |
| **White** | **PPV** | **INPC** | 0.987 | 0.983 – 0.990 | **>65 years** | **PPV** | **INPC** | 0.985 | 0.980- 0.990 |
|  | **PPV** | **NBS** | 0.895 | 0.887- 0.903 |  | **PPV** | **NBS** | n/a | n/a |
|  | **PPV** | **SSA** | n/a | n/a |  | **PPV** | **SSA** | 0.989 | 0.985- 0.993 |
|  | **PPV** | **MCHD** | n/a | n/a |  | **PPV** | **MCHD** | 0.982 | 0.967 -0.993 |
| **White** | **F-score** | **INPC** | 0.988 | 0.9850.990 | **>65 years** | **F- score** | **INPC** | 0.982 | 0.977- 0.985 |
|  | **F-score** | **NBS** | 0.877 | 0.870- 0.883 |  | **F- score** | **NBS** | n/a | n/a |
|  | **F-score** | **SSA** | **n/a** | **n/a** |  | **F- score** | **SSA** | 0.855 | 0.845 -0.865 |
|  | **F-score** | **MCHD** | n/a | n/a |  | **F- score** | **MCHD** | 0.974 | 0.962-0.985 |
| **Hispanic** | **Sensitivity** | **INPC** | 0.980 | 0.955-1.00 | **Male** | **Sensitivity** | **INPC** | 0.971 | 0.965- 0.977 |
|  | **Sensitivity** | **NBS** | 0.858 | 0.834-0.884 |  | **Sensitivity** | **NBS** | 0.860 | 0.850- 0.871 |
|  | **Sensitivity** | **SSA** | n/a | n/a |  | **Sensitivity** | **SSA** | 0.764 | 0.750- 0.779 |
|  | **Sensitivity** | **MCHD** | n/a | n/a |  | **Sensitivity** | **MCHD** | 0.944 | 0.934- 0.953 |
| **Hispanic** | **PPV** | **INPC** | 0.980 | 0.955-1.000 | **Male** | **PPV** | **INPC** | 0.965 | 0.957-0.971 |
|  | **PPV** | **NBS** | 0.906 | 0.884-0.926 |  | **PPV** | **NBS** | 0.908 | 0.900-0.918 |
|  | **PPV** | **SSA** | n/a | n/a |  | **PPV** | **SSA** | 0.988 | 0.984 – 0.993 |
|  | **PPV** | **MCHD** | n/a | n/a |  | **PPV** | **MCHD** | 0.979 | 0.973 -0.985 |
| **Hispanic** | **F-score** | **INPC** | 0.980 | 0.962- 0.994 | **Male** | **F-score** | **INPC** | 0.968 | 0.963- 0.972 |
|  | **F-score** | **NBS** | 0.882 | 0.864- 0.899 |  | **F-score** | **NBS** | 0.884 | 0.876-0.891 |
|  | **F-score** | **SSA** | n/a | n/a |  | **F-score** | **SSA** | 0.862 | 0.852 – 0.872 |
|  | **F-score** | **MCHD** | n/a | n/a |  | **F-score** | **MCHD** | 0.961 | 0.955-0.967 |
| **Not Hispanic** | **Sensitivity** | **INPC** | 0.991 | 0.988- 0.994 | **Female** | **Sensitivity** | **INPC** | 0.979 | 0.975- 0.983 |
|  | **Sensitivity** | **NBS** | 0.860 | 0.850- 0.869 |  | **Sensitivity** | **NBS** | 0.862 | 0.850 0.874 |
|  | **Sensitivity** | **SSA** | n/a | n/a |  | **Sensitivity** | **SSA** | 0.809 | 0.794- 0.823 |
|  | **Sensitivity** | **MCHD** | n/a | n/a |  | **Sensitivity** | **MCHD** | 0.953 | 0.946- 0.961 |
| **Not Hispanic** | **PPV** | **INPC** | 0.991 | 0.988-0.994 | **Female** | **PPV** | **INPC** | 0.978 | 0.973- 0.982 |
|  | **PPV** | **NBS** | 0.884 | 0.876 – 0.892 |  | **PPV** | **NBS** | 0.900 | 0.890- 0.909 |
|  | **PPV** | **SSA** | n/a | n/a |  | **PPV** | **SSA** | 0.989 | 0.984- 0.993 |
|  | **PPV** | **MCHD** | n/a | n/a |  | **PPV** | **MCHD** | 0.983 | 0.978- 0.988 |
| **Not Hispanic** | **F-score** | **INPC** | 0.991 | 0.989-0.993 | **Female** | **F-score** | **INPC** | 0.978 | 0.975- 0.981 |
|  | **F-score** | **NBS** | 0.872 | 0.865-0.879 |  | **F-score** | **NBS** | 0.881 | 0.872-0.888 |
|  | **F-score** | **SSA** | n/a | n/a |  | **F-score** | **SSA** | 0.890 | 0.880- 0.899 |
|  | **F-score** | **MCHD** | n/a | n/a |  | **F-score** | **MCHD** | 0.968 | 0.964-0.972 |

**Table S1.** Matching performance parameters, stratified by sociodemographic groups across all datasets. PPV denotes Positive Predictive Value. INPC = Indiana Network for Patient Care; NBS = Newborn Screening; SSA = Social Security Administration; MCHD = Marion County Health Department; n/a = not available
